# Effects of a Six-Week International Tour on the Physical Performance and Body Composition of Young Chilean Tennis Players

**DOI:** 10.3390/ijerph20021455

**Published:** 2023-01-13

**Authors:** Pablo Luna-Villouta, Marcelo Paredes-Arias, Carol Flores-Rivera, Claudio Hernández-Mosqueira, Jaime Vásquez-Gómez, Carlos Matus-Castillo, Rafael Zapata-Lamana, César Faúndez-Casanova, Néstor Jofré Hermosilla, Natalia Villar-Cavieres, Rodrigo Vargas-Vitoria

**Affiliations:** 1 Facultad de Educación, Pedagogía en Educación Física, Universidad San Sebastián, Concepción 4030000, Chile; 2 Escuela de Salud, Técnico Superior en Preparación Física, Instituto Profesional Duoc UC, Puente Alto 8190777, Chile; 3 Facultad de Educación y Ciencias Sociales, Universidad Andres Bello, Concepción 4030000, Chile; 4 Departamento de Educación Física, Deportes y Recreación, Universidad de La Frontera, Temuco 4780000, Chile; 5 Centro de Investigación de Estudios Avanzados del Maule (CIEAM), Universidad Católica del Maule, Talca 3460000, Chile; 6 Laboratorio de Rendimiento Humano, Universidad Católica del Maule, Talca 3460000, Chile; 7 Departamento de Ciencias del Deporte y Acondicionamiento Físico, Universidad Católica de la Santísima Concepción, Concepción 4030000, Chile; 8 Escuela de Educación, Universidad de Concepción, Los Ángeles 4440000, Chile; 9 Facultad de Educación, Pedagogía en Educación Física, Universidad Católica del Maule, Talca 3460000, Chile; 10 Escuela de Kinesiología, Facultad de Salud, Universidad Santo Tomás, Concepción 4030000, Chile; 11 Departamento de Formación Inicial Escolar, Universidad Católica del Maule, Talca 3460000, Chile

**Keywords:** body composition, physical performance, tennis, tour

## Abstract

In tennis, it is common for young male tennis players to spend several weeks away from their local training camps during the competition season, which could affect their performance. The purpose of the study was to analyze the effects of a six-week international tour on physical performance and body composition in young Chilean tennis players. Twenty-four men between the ages of 14 and 16 participated in this research. In body composition and anthropometric measurement, body weight, height, skinfolds, and perimeters were measured. Body fat percentage (BFP) and skeletal muscle mass (SMM) were calculated. For physical performance, 5-m and 10-m sprints, modified agility test (MAT test), countermovement jump (CMJ), and medicine ball throw (MBT) were evaluated. Results show that, in body composition, BFP and SMM significantly decreased post-tour (*p* < 0.05; effect sizes ranging from 0.23 to 0.33, respectively). In physical performance, agility and 5-m and 10-m sprints significantly decreased (*p* < 0.05, effect sizes ranging from −0.63 to 1.10). We conclude that after a six-week international tour, BFP, SMM, agility, and speed (linear sprint) tend to decrease significantly, with a greater effect in the sprint tests.

## 1. Introduction

Tennis is a dynamic and explosive sport, which demands a display of high power and speed when hitting and moving around the court, which alternates short and intermittent high-intensity efforts with rest periods [1]. This leads to inferring a load vs. rest ratio from 1:1 to 1:5 [2]. A point in tennis lasts approximately 8 to 10 s, with a mean of 10 strokes with four changes of direction, with sprints between 2 and 6 m [3]. The total duration of a match is, on average, 1.5 h for match of 2 or 3 sets and the percentage of effective playing time is 20% to 30% [4]. This shows that tennis is a sport with a high physical load [5,6,7], where the player must respond to a high and changing number of efforts and competition loads (between 300 and 500), having to endure long periods of time with intermittent high-intensity efforts with different movements at the level of the upper body (strokes) and lower body (movements on the court) [8]; therefore, a very good amount of preparation and a high level of physical performance are required to successfully respond to the demands of the game [9].

Young tennis players usually participate in consecutive matches in one day, either in singles or doubles, as part of their usual competition schedule [10], which means that the physical demand in training and competitions is often very high with a short recovery span [11]. This can result in tennis players having inadequate recovery time with an increased risk of injury [12] and a decrease in physical performance [10]. In addition, it is common that throughout the competition season, young tennis players are away from local facilities for several weeks and without direct supervision of their trainers, which could reduce the effects of individual training and their performance [13].

In general, in tennis, studies on prolonged and repeated play have found a decrease in different parameters of physical and technical performance, such as a decrease in muscular strength [10,14,15], decrease in speed [10,15], and serve speed [10,15,16], and even losses in stroke accuracy [11,14].

In addition, a decrease in 5-m, 10-m, and 20-m sprints has been reported in young tennis players after a five-week international tour [17]. It has also been shown that five weeks of unsupervised training decreases speed, power, and aerobic capacity in college tennis players [13].

Due to the importance of physical performance in tennis, load control and recovery can provide valuable information for the optimal design of training and injury prevention [12,18]. In fact, body composition plays an important role in the performance of sports where the speed of acceleration and braking, together with the ability to generate high levels of force are determinants for success in this sport [19].

Based on this background, it is possible to observe that the study of the effect of repeated play on young tennis players is very important, but it is still limited, so much so that, in South America, we did not find research on the subject, we only found some anthropometric characteristics [20,21,22,23,24], cross-sectional evaluations of physical performance [7,25], and mental characteristics [26,27].

As indicated above, the hypothesis of this research is that a six-week international tour decreases physical performance and modifies body composition in young tennis players.

Therefore, the main objective of this study was to analyze the effects of a six-week international tour on physical performance and body composition in young Chilean male tennis players. As a secondary objective, we sought to analyze the modifications of physical performance and body composition by national ranking, for which two groups were established: “national ranking 1–20” (players ranked between number 1 to 20) and “national ranking 21–50” (players ranked between number 21 to 50).

## 2. Materials and Methods

### 2.1. Participants

To gather data for the current observational cross-sectional study, 24 Chilean male tennis players between the ages of 14 and 16 from sports clubs in Santiago (Chile) were considered for descriptive analysis. Participation was voluntary and evaluations were realized a week before (pre-tour) and a week after (post-tour) during a six-week international tour competition for junior male tennis players organized by the South American Tennis Confederation (COSAT), in the countries of Argentina (2 weeks), Uruguay (1 week), Paraguay (1 week), and Brazil (2 weeks). In each tournament, the players played 4 to 7 matches in the singles category (media = 5.5) on clay tennis courts. The sample size was n = 24 and the inclusion criteria were: (1) being a Chilean male tennis player between 14 and 16 years old; (2) competition should include international tournaments in the last 12 months; (3) ranking in Chile should span between 1 and 50; and (4) complete six-week COSAT international tour. The exclusion criteria were: (1) failure to complete all the evaluations; (2) failure to appear with appropriate clothing or sports sneakers for physical evaluations; and (3) physical injury that affects the results of the evaluations. G*Power version 3.1.9.7 (Heinrich-Heine-Universität Düsseldorf, Düsseldorf, Germany) was used to process sample running a single-sample t-test which showed a bilateral alpha error of 5%; effect size 0.75 and statistical power of 80%.

For the data collection process, authorization was requested from the directors of the tennis clubs in written form, where information about the objective and tests to be applied were described. Once the clubs accepted, consent forms were given to the player’s parents, informing them about the objective of the research and characteristics of the evaluations. After approval and signing, subjects’ participation in the evaluations was confirmed, according to the Declaration of Helsinki, updated at the World Medical Assembly in Fortaleza (2013) for human research [28]. In addition, the study was approved by a competent ethics committee in the academic field, Ethics Committee of Universidad San Sebastián, Chile (USS 51-2018-20, 2019).

### 2.2. Procedures

All anthropometric evaluations were performed individually in the morning before any type of exercise, in a private and specially equipped room. Following the standard procedures of Marfell-Jones et al. 2012 [29], measurements were taken by an experienced evaluator. Height (cm) was measured (without shoes) in the Frankfurt plane, with an aluminum stadiometer graduated in millimeters (Seca 220, Hamburg, Germany). Body weight was checked with a mechanical scale (Seca 700, Hamburg, Germany), with a measurement of 50 g and ranged from 0 to 220 kg. Skinfolds were measured in triceps brachial, anterior thigh, and medial leg with an anthropometric caliper (Harpenden ^®^, Baty International Ltd., West Sussex, UK). Arm, thigh, and leg perimeters were measured with a Lufkin^®^ Metallic anthropometric tape (Medina, OH, USA). All anthropometric variables were measured three times. The technical error of measurements ranged from 0.25% to 0.8%.

Percent body fat was obtained using the equation (BFP = 0.735 (triceps + calf) + 1.0) proposed by Slaughter et al. (1988) [30], skeletal muscle mass (SMM) was calculated with the equation by Poortmans et al. (2005) [31], SMM (kg) = height × ((0.0064 × corrected circumference for upper arm^2^) + (0.0032 × corrected circumference for thigh^2^) + (0.0015 × corrected circumference for calf^2^) + (2.56 × sex) + (0.136 × age)). Biological maturation was calculated by age at peak height velocity (APHV), using the equation by Moore et al. (2015) [32], (Maturity offset = −7.999994 + (0.0036124 × (age × height)).

All physical performance tests were made on clay tennis courts during the morning following anthropometric evaluations. Participants wore athletic clothing (shorts, shirt, and tennis shoes). Evaluations were carried out by two experienced evaluators (both with Masters in Sports Sciences, MSc.) previously trained theoretically and practically in administering the tests. Testing was done before and after international tour where each test was carried out twice per individual, with a 2- to 5-min break between tests. The best values of the day were recorded. The test itself started with a 15-min warm-up (general physical exercises and stretching). The application structure was as follows. First, for the sprint test (5-m and 10-m sprints), the player had to stand behind the starting line and, at the signal, run the indicated distance as fast as possible. Second was the MAT test (Modified Agility Test), where the player was compelled to move and change direction over a total distance of 20 m. Four cones were arranged in a “T” shape for the player to sprint in a straight line to the first cone placed at 5 m, and then, laterally without crossing feet, towards a second cone 2.5 m to his left. The player then turned to the right side to reach the third cone, located at 5 m. Then, the player should return to the middle cone and the test ends when they reach the starting position. To ensure form would not to be compromised, cones were touched every time that position was reached. Sprints and agility tests were recorded with a manual digital stopwatch. Third, the medicine ball throw was performed standing and with one hand (MBT), the throw was executed by the side of the head with a 2 kg ball and with the player’s preferred hand. The distance was measured with a Stanley Power Lock millimeter tape (USA). Finally, countermovement jumps (CMJ) were done, which were measured with a Globus Ergo Jump platform (Bosco System), according to the protocol of Bosco and Padulles (1994) [33].

### 2.3. Statistical Analysis

Statistical analysis was performed using 17.0 SPSS IBM Corp. (IBM®, Somers, NY, USA) and GraphPad Prism 7.0 software (GraphPad®, San Diego, CA, USA). The values obtained are shown with descriptive statistics of mean and standard deviation (SD). The Shapiro–Wilk test determined the normal distribution of the variables. The differences between pre- and post-tour measurements was determined by *t*-test for related samples. Additionally, the effect size (ES) of the changes in each variable was calculated with Cohen’s d, interpreted as follows: 0.2 (small), 0.5 (moderate), and 0.8 (large) [34]. The significance level used was *p* < 0.05.

## 3. Results

Table 1 shows the values of mean, SD, *t*-test, and effect size given by Cohen’s d for comparison between pre- and post-tour tests in body composition and anthropometric measures. In general, post-competition values are lower in BW, SMM, ∑ 3 Skinfolds, and BFP, with significant decreases post-tour (*p* < 0.05), and “moderate” ES (d = 0.23 to 0.48).

Table 2 presents the values of mean and SD of the *t*-test and effect size given by Cohen’s d, for comparison between pre- and post-tour physical performance tests. In the post-tour tests, the group shows a significant decrease in 5-m, 10-m sprints, and agility (*p* < 0.05), with ES “large” in sprint tests (5_m d = 1.10; 10_m d = −0.92) and “moderate” in agility (d = −0.63). On the other hand, in CMJ and MBT, there were no significant differences between pre- and post-tour (*p* > 0.05).

**Figure 1:**
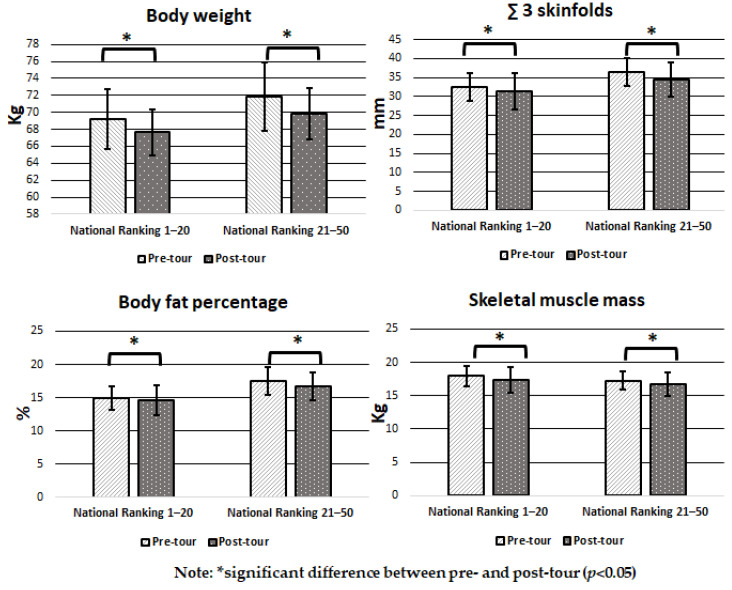
Mean and SD in pre- and post-tour body composition and anthropometric measures by Chilean junior tennis ranking.

Figure 1 shows the comparison in body composition and anthropometrics measures pre- and post-tour for the “national ranking 1–20” and “national ranking 21–50” groups. The two groups present a significant decrease post-tour (*p* <0.05) in BW, ∑ 3 Skinfolds, SMM, and BFP.

**Figure 2:**
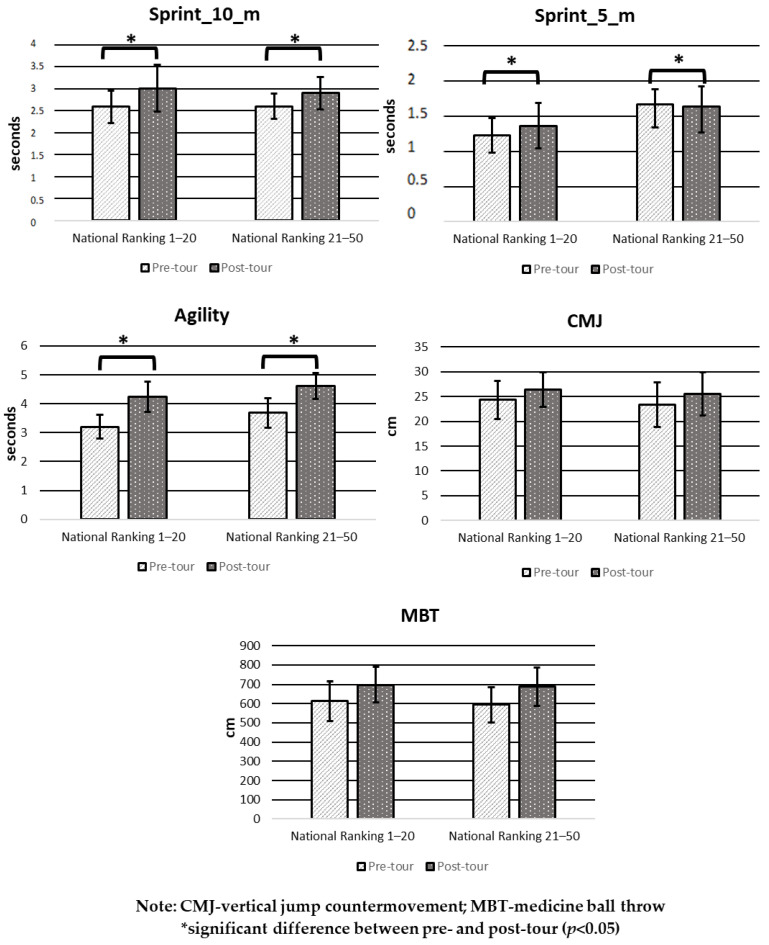
Mean and SD in pre- and post-tour physical performance tests by Chilean junior tennis ranking.

Figure 2 shows the comparison in the pre- and post-tour physical performance tests for the “national ranking 1–20” and “national ranking 21–50” groups. Both groups show a similar behavior in physical performance, with a significant decrease after competition in 5-m, 10-m sprints, and agility (*p* < 0.05). In contrast, in CMJ and MBT, there were no significant differences between pre- and post-tour tests (*p* > 0.05).

## 4. Discussion

The study aimed to analyze the effects of a six-week international tour on physical performance and body composition in young Chilean tennis players. The results show that, in the variables of physical performance, after the international tour, there was a significant decrease (*p* < 0.05) in agility and sprint (5-m and 10-m), ES of the changes was “moderate” in agility (d = −0.63) and “large” in sprint tests (5_m d = 1.10; 10_m d = −0.92). On the other hand, body composition showed significant decreases in BW, ∑ 3 Skinfolds, BFP and SMM (*p* < 0.05), with “moderate” ES (d = 0.23 to 0.48).

When analyzing the changes in physical performance and body composition by national ranking, the two groups (“national ranking 1–20” and “national ranking 21–50”) showed post-tour significant decreases (*p* < 0.05) in both the body composition and physical performance, therefore the negative effects of long-term travel and competitions away from local facilities occur regardless of the level of sports performance of young tennis players. Negative changes in performance due to lack of coach supervision and leaving training facilities for similar periods have also been observed in other studies with tennis players [13,17]. Coaches should implement strategies to maintain fitness between tournaments, particularly in linear sprint, as highlighted above [17].

The significant decrease in 5-m and 10-m sprints coincides with that reported in another study in young tennis players after a five-week international tour [17], and for playing consecutive matches on the same day [10]. In addition, this negative effect was described for 5-m sprint in adult tennis players after matches on consecutive days [15]. In this regard, it is important to bear in mind that the speed of movement is an important measure of performance in different sports modalities, and that in tennis, it allows the athlete to comply with the game plan and better position himself to hit the ball [35]. Therefore, it seems important to properly monitor their condition and specific training [36] considering that the reduction in speed will make it difficult for competitive tennis players to reach their optimal potential [13].

Agility also showed a significant decrease after the tour, although the ES of this change was “moderate”. The decrease in agility has been reported when analyzing the effects of two consecutive matches in young tennis players [10], but they have not been detected in other studies in young tennis players [17] or in university tennis players [13] where they found no significant modifications in agility. This may be because the movement patterns evaluated in agility test are movements very similar to those typical of the game of tennis, which may be an adequate stimulus and thus avoid large losses of this quality [17]. We believe that this happens since the player, during each game, must be changing position constantly and is mostly moving at a high speed in different directions and with different movements (lateral and frontal displacements, jumps, turns, etc.) [37].

In CMJ and MBT, there were no significant reductions in muscle power tests; other studies in tennis players have also reported no changes in this type of tests [13,15,17,38]. One possible explanation is that unique explosive movements, such as jumps or throwing appear to be less affected by disruption of specific training over a six-week period [39]. Another possible answer is that tennis, being a sport where a high number of high-intensity movements are repeated at the level of upper body (strokes) and lower body (displacements on the court), is sufficient to maintain muscle power levels [13].

In physical performance, the results show that the preparation for many weeks to get ready for international tours should focus on neuromuscular, plyometric, and movement training at maximum intensity. This can avoid detected decrease in agility and sprint and consequently maintain or improve physical performance and quick (re) actions in young players, helping to satisfactorily meet competition demands [3,10]. This type of exercise should also be included during tournaments and international tours, whenever time slots in between matches and championships may allow. In addition, its compliance by tennis players can be remotely monitored through software applications on mobile devices such as smartphones or smartwatches, which would facilitate the work of coaches not travelling with the players.

The body composition measures showed significant differences between pre- and post-tour values in BW, ∑ 3 Skinfolds, SMM, and BFP; although the ES of these changes was only “moderate”, this does not coincide with another five-week study of unsupervised training in university tennis players, where no significant decreases were found in the body composition [13]. In this aspect, the information seems to be contradictory, since there are studies in other population groups and sports that do not indicate significant differences in fat-free mass or body fat percentage product of similar periods of training [40,41], and, on the contrary, others indicate significant changes, both in BFP and SMM [39,42,43]. However, the influence of training and competition on body composition modifications, both in SMM and BFP have been reported for different sports, highlighting that these modifications are influenced by sports and physical activity in young boys [44]. These changes in body composition have notorious positive effects for healthy development, physical performance, and injury prevention in young athletes [45].

The modifications found in the body composition reinforce the importance of adequate nutrition and recovery in between matches and competitions. In this regard, recommendations aim to educate tennis players in following nutritional guidelines, hydration, and recovery methods between each match in tournaments [10,11,15]. In addition, it is recommended that during trips, simple anthropometric measurements should be made periodically, such as control of body weight and body perimeters, which can be self-applied by the players, using small and easy-to-carry materials such as an anthropometric tape. Currently, all these recommendations and measurements are easy to monitor remotely through software applications on mobile device. This study has some limitations, such as the small number of subjects evaluated. Another limitation derives from the homogeneity in the characteristics and level of competition of the evaluated tennis players, which could limit the use of the results in other competitive levels, other disciplines, or ethnic groups. The strengths of the study are based on the lack of research on this subject in South American tennis players; therefore, these results can provide important evidence on the effect of international tours on the performance of young tennis players. Reliable and valid procedures and tests were used which were simple and fast to administer with low economic costs and can be easily replicated

## 5. Conclusions

It is concluded that after a six-week international tour, young Chilean tennis players significantly decrease body composition (BFP and SMM), agility, and 5-m and 10-m sprints, with greater effect on sprint tests. Based on these results, preparation for international tours should strengthen speed and agility training, as well as provide adequate nutritional strategies and neuro-muscular recovery between matches and tournaments, which can help avoid reductions in performance and the risk of injury or alterations in the health of young tennis players.

## Figures and Tables

**Table 1:** Pre- and post-tour body composition and anthropometric measures.

Variables	Pre-Tour (n *=* 24)		Post-Tour (n *=* 24)					
	Mean	SD	Mean	SD	*p*-Value	% Difference	ES	
							*d*	Qualitative
Age (years)	14.9	0.6	15.0	0.6	0.352	0.9	−0.27	moderate
APHV (levels)	1.2	0.3	1.3	0.3	0.230	3.8	−0.24	moderate
Height (cm)	171.4	2.9	171.6	2.8	0.822	0.1	−0.06	small
BW (kg)	70.5	3.3	68.8	3.7	0.001 *	−2.5	0.48	moderate
SMM (kg)	17.6	1.8	17.0	1.6	0.001 *	−3.1	0.33	moderate
∑ 3 skinfolds (mm)	34.4	4.7	32.9	4.4	0.001 *	−4.3	0.28	moderate
BFP (%)	16.2	2.4	15.7	2.3	0.001 *	−3.3	0.23	moderate
Training hours/week	24.9	4.9	24.6	5.1				

Note: APHV—peak growth rate acceleration; BW—body weight; SMM—skeletal muscle mass; BFP—body fat percentage. * significant difference (*p* < 0.05).

**Table 2:** Pre- and post-tour physical performance tests.

Variables	Pre-Tour (n *=* 24)		Post-Tour (n *=* 24)					
	Mean	SD	Mean	SD	*p*-Value	% Difference	ES	
							*d*	Qualitative
Sprint 5-m (s)	1.3	0.2	1.7	0.3	0.001 *	30.1	−1.10	large
Sprint 10-m (s)	2.6	0.3	3.0	0.5	0.003 *	13.8	−0.92	large
Agility (s)	3.7	0.7	4.1	0.7	0.035 *	11.9	−0.63	moderate
CMJ (cm)	25.4	4.2	24.5	4.0	0.446	−3.4	0.22	moderate
MBT (cm)	655.4	104.2	640.8	106	0.792	−2.1	0.13	small

Note: CMJ—vertical jump countermovement; MBT—medicine ball throw. * significant difference (*p* < 0.05).

## Data Availability

The data presented in this study are available on request from the corresponding author. The data are not publicly available due to ethical considerations of the human study.
